# Evaluation of the Effect of Nutrition Education on Osteopathic Medical Students’ Personal Nutrition Choices and Incorporation Into Future Medical Practice

**DOI:** 10.7759/cureus.66645

**Published:** 2024-08-11

**Authors:** Amanda T Parker, Robert Bateman, Danielle Fastring

**Affiliations:** 1 Osteopathic Medicine, William Carey University College of Osteopathic Medicine, Hattiesburg, USA; 2 Student Research/Preclinical Sciences, William Carey University College of Osteopathic Medicine, Hattiesburg, USA

**Keywords:** curriculum development and evaluation, medical education curriculum, medical student health, osteopathic education, nutrition education

## Abstract

Context: Our current research project evaluates the impact of nutrition education on the medical student’s personal nutrition goals and the likelihood of incorporating nutrition needs into patient evaluation and treatment plans in future practice. The growing popularity of lifestyle medicine has further emphasized the importance of nutrition in the treatment of all patients, especially those suffering from chronic diseases. The paucity of formal medical nutrition education in medical school curricula leaves a significant gap in the knowledge base of physicians in practice.

Objective: In an attempt to close the gap, we increased nutrition education in first-year osteopathic medical students by establishing a nutrition course emphasizing modern competencies and their importance in clinical practice.

Methods: The course evaluation utilized a two-group quasi-experimental pre-test/post-test study design. The intervention group consisted of medical students participating in the newly established course, and the control group consisted of second-year medical students who had not taken the course as part of their curriculum. Information was collected about students’ knowledge, attitudes, behaviors around nutrition, their plans to pursue a residency with a focus on culinary medicine and incorporate medical nutrition into their medical practice in the future, and their intention to incorporate culinary medicine into future treatment plans. Participation rates within a voluntary culinary medicine interest group were also tracked.

Results: Students in the intervention group were 26% more likely to report that proper nutrition for patient care had been addressed in coursework. This suggests that medical students exposed to the medical nutrition course have received more training that is critical in proper patient care than students in the control group. Students in the intervention group were 93% more likely to believe that proper nutrition can be used to prevent disease. Lastly, significantly more students in the intervention group (33% more) intend to explore residency programs with a focus on culinary medicine than those students in the control group.

Conclusion: These results demonstrate that nutritional education promotes an awareness of the effectiveness of nutritional counseling in disease prevention and management. Furthermore, it will hopefully prompt future physicians to consider nutritional counseling during their clinical rotations, through residency, and into their independent practice. The presentation of nutrition in the first year of medical school is critical to develop increasing numbers of primary care physicians that promote the importance of nutrition and a healthy lifestyle for patients.

## Introduction

Nutrition plays a central role in a healthy lifestyle [1]. Unhealthy diets and improper nutrition contribute to the development of chronic diseases such as metabolic syndrome, cardiovascular disease, various cancers, and even neurological disorders on a global scale [2]. This effect is apparent in the relentless increase in the percentage of overweight and obese members of the population. According to the National Health and Nutrition Examination Survey Statistics Reports from 2015 to 2018, the percentage of Americans 20 years of age and older considered overweight or obese reached 72.5% [3]. Reported by the World Health Organization, obesity rates have doubled globally since 1990 resulting in a staggering 1 billion people in the world living with obesity [4]. As a consequence, record increases in diabetes, hypertension, heart disease, and other modifiable diseases are plaguing the healthcare field. Evidence of the growing impact of nutrition on health was demonstrated by the recent White House Conference on Hunger, Nutrition and Health. This monumental conference marked the first discussion of nutrition and health on this level in over 50 years. With the increased correlation between nutrition and health, physicians are in a unique position to impact patient health through nutrition education but are often failing to do so due to obstacles such as incomplete nutrition education, [5] lack of time with patients, [6] and current reimbursement procedures [7].

In a comprehensive review of the state of U.S. health in JAMA, poor diet is identified as a serious risk that warrants increased attention due to its negative effect on national health [8]. Evaluation of the literature, however, demonstrates a gap in the nutrition content of medical education. In 1985, the National Academy of Sciences recommended a minimum of 25 hours of nutrition education be provided by medical schools [9]. Further emphasis on the importance of nutrition knowledge in the medical school curriculum was noted by the addition of the nutrition subscore in the United States Medical Licensing Examination (USMLE) reporting in 2003. However, studies demonstrate that the actual inclusion of nutrition education in medical curricula varies among institutions. A 2006 study noted nutrition education received by medical students remained inadequate [10]. An evaluation of nutrition education among schools of osteopathic medicine found that 85% do not meet the recommended minimum of 25 hours [11] demonstrating a consistent gap in the medical curricula. To improve the nutrition content in medical education, the International Association of Medical Science Educators (IAMSE) committee identified learning objectives needed in medical school curricula [5]. Even with identified objectives, the majority of medical schools in the United States continue to lack sufficient nutrition content within the curriculum [12].

This national discussion has also had an impact on medical education in that those responsible for curriculum development are seeing the importance of including nutrition education in the proper training of medical professionals. The inclusion of nutrition-focused medical education has led to increased utilization of terms like lifestyle medicine and culinary medicine [13]. Lifestyle medicine incorporates the importance of nutrition and other factors such as physical activity, sleep, mental health, avoiding harmful substances, healthy relationships, and social determinants of health into the treatment plan of patients. The American College of Lifestyle Medicine (ACLM) was established in 2004 as a society that aims to increase quality education and certification for medical professionals seeking to improve patient care through implementing lifestyle changes. Currently, ACLM offers a variety of curricula for implementation in medical schools, health profession education programs, and residency programs. In addition, the ACLM offers continuing education credits for practicing physicians as well as certification. Culinary medicine has been implemented in numerous medical schools, residency programs, and nursing schools to enhance the training of medical professionals in the use of food as medicine in various forms including being offered as third- or fourth-year electives. Culinary medicine specifically addresses the preparation and delivery of healthy meals.

As defined by the American Osteopathic Association, osteopathic medicine utilizes the self-healing ability of the body and promotes overall wellness to prevent the development of disease. Effective nutrition training is a natural addition to an osteopathic curriculum and is offered at select osteopathic medical schools as an elective. With the holistic nature of osteopathic medicine as well as the high percentage of osteopathic physicians that enter the primary care specialties, explicit instruction in nutrition and other aspects of lifestyle medicine fit naturally into their training.

The paucity of formal medical nutrition education in medical school curricula leaves a significant gap in the knowledge base of physicians in practice. In an attempt to close the gap, we developed a mandatory nutrition course emphasizing modern competencies and relevance to clinical practice. Initial course competencies in year one utilized the core nutrition competencies [5] with consultation from two registered dietitians. The nutrition course provides invaluable interprofessional training for future physicians. Using a uniquely qualified instructor and guest lecturers, our students are exposed to the interprofessional approach of inviting participation from dietitians and other health professionals to properly utilize food as medicine. Multiple course topics within our course are presented by a certified dietician who is also an osteopathic physician. Our cross-trained instructor provides the students with dietitian recommendations for patients in various disease states, primary critical care nutrition planning, motivational interviewing skills, and a discussion of the use of a dietitian referral. In addition, the course featured a physician guest lecturer who demonstrated the proper assessment of nutritional status in a standard clinical encounter. Lastly, we invited an exercise physiologist to provide instruction around encouraging patients to increase their physical activity.

As we increased nutrition education at William Carey University College of Osteopathic Medicine, we also wanted to determine the effect of this nutrition course on attitudes of first-year medical students as part of our continued commitment to evaluating curricular changes. This project aims to evaluate the impact of the implementation of more nutrition education on medical students’ personal nutrition goals and the likelihood of incorporating nutrition needs in patient evaluation and treatment plans into future practice. In addition, this research project will assess the need for further incorporation of comprehensive lifestyle medicine education in our osteopathic medical school curricula to improve the effectiveness of physicians in our community.

Objectives

The primary objective of this study is to evaluate the impact of a mandatory nutrition course on the attitudes and practices of first-year medical students at William Carey University College of Osteopathic Medicine. We aim to assess how the course influences students' personal nutrition goals and their likelihood of incorporating nutrition into patient care. Furthermore, this research seeks to identify the need for further integration of lifestyle medicine education within the osteopathic medical curriculum. Initial research will provide insight into the immediate impact on students, while plans to follow their career paths will allow for tracking the long-term effects of nutrition education.

## Materials and methods

A quasi-experimental, pre-test /post-test design was used to assess differences in personal behaviors related to nutrition, intention to incorporate nutrition needs into patient evaluation and treatment plans into future practice, and the impact that medical school had on those behaviors and intentions. The intervention group consisted of first-year osteopathic medical students enrolled in a mandatory nutrition course. The control group included second-year osteopathic students who did not receive the nutrition course as part of their year 1 osteopathic medical school curriculum. The study protocol was approved by the William Carey University Institutional Review Board and deemed exempt (IRB #2021-008). All students provided informed consent prior to participation.

All survey questionnaires were created by the authors and can be found in Appendices. Surveys were distributed electronically via email to 181 students, 54 in a control group and 127 in an intervention group, through the Qualtrics survey platform. Out of the 181 students who were sent the survey, 125 completed both the pre- and post-tests; therefore, data analysis was conducted using these 125 responses. Categorical demographic variables were analyzed using IBM SPSS Statistics for Windows, Version 27 (Released 2020; IBM Corp., Armonk, New York, United States) and reported as frequencies and percentages within each category. To determine if the intervention and control groups differed with regard to demographics at baseline, a chi-square analysis was conducted. Longitudinal variables were analyzed and reported with mean and standard deviation or median and range as appropriate. An independent t-test was utilized to determine if the intervention and control groups differed significantly with regard to age. At post-test, chi-square analysis was conducted to determine if the intervention and control groups differed significantly in their level of agreement with each statement presented.

In order to assess individual readiness to make a nutritionally related behavioral change, Prochaska’s Transtheoretical Model, otherwise known as the Stages of Change Model, was utilized [14]. The theory has been applied to many health behaviors and has shown that people who are successful at making behavioral and lifestyle changes move through five distinct stages. The five stages are precontemplation (no intention to change behavior, unaware of the need for behavioral change), contemplation (aware that there is a need to change, but do not have a plan to take action), preparation (individuals make a plan to change behavior, but are not implementing the plan, action (individuals are implementing behavioral changes, maintenance (you have been successful in continuing the action phase for six months or more). This framework was utilized to determine the stage of behavioral change (readiness) around nutrition improvement of first-year osteopathic medicine students participating in the course.

As part of the standard procedures for course evaluation at the University, students completed end-of-course evaluations. In addition, focus groups composed of members of the class were conducted to obtain detailed student feedback. Student feedback will be utilized to make improvements to the course.

## Results

Participants who completed the baseline survey (n=181) provided demographic information (Table 1).

**Table 1:** Demographic Characteristics of Participants at Baseline (n=181) n: sample size; %: percent of individuals in each category; X̅: mean; SD: standard deviation For categorical variables, * denotes the significance of the chi-square test comparing the control group to the intervention group. For age, * denotes the significance of the independent t-test comparing the control group to the intervention group.

Demographic	Control (n=54)	Intervention (n=127)	Significance p <0.05*
	n (%)	n (%)	
Race			
Caucasian	25 (46.3)	66 (52.0)	0.229
South Asian	13 (24.1)	30 (23.6)	
Southeast Asian	4 (7.4)	2 (1.6)	
Middle Eastern	2 (3.7)	5 (3.9)	
East Asian	1 (1.9)	12 (9.4)	
Other	3 (5.6)	4 (3.1)	
Prefer not to Answer	4 (7.4)	4 (3.1)	
Ethnicity	n (%)	n (%)	
Hispanic	2 (3.7)	8 (6.3)	0.493
	X̅ ± SD	X̅ ± SD	
Age	26.9±3.60	25.7±3.1	0.529

There were no significant differences between participants in the intervention group (n=127) and control group (n=54) at baseline.

The post-test was completed by 125 students. Twenty-five were in the control group, and one hundred were in the intervention group. There were three areas in which participants were found to be statistically significantly different at post-test. These were as follows: (i) Proper nutrition for patient care has been addressed in my coursework to this point: In the control group, 64.0% ‘somewhat agreed’ or ‘strongly agreed’ with the statement whereas in the intervention group 90.0% ‘somewhat agreed’ or ‘strongly agreed’ (Χ^2^ =15.5, p<0.004, df(degrees of freedom) =4). These data demonstrate that medical students exposed to the medical nutrition course noted an increase in the level at which proper nutrition for patient care had been addressed in coursework. This suggests that medical students exposed to the medical nutrition course have been exposed to more training that is critical in proper patient care; (ii) The belief that proper nutrition can be used to prevent disease: In the control group, 0.0% ‘somewhat agreed’ or ‘strongly agreed’ with the statement whereas in the intervention group 93.0% ‘somewhat agreed’ or ‘strongly agreed’ (Χ^2^=9.86, p<0.043, df=4). These data demonstrate the increase in the importance of educating patients on proper nutrition to prevent disease. In addition, data analysis revealed students exposed to the nutrition course noted that medical school curricula positively affected personal fitness and health goals suggesting that the mandatory nutrition and lifestyle medicine course positively affected the medical student’s readiness to engage in behavioral change around personal nutrition goals; (iii) I plan to complete culinary medicine certification following residency: In the control group, 51.0% ‘somewhat agreed’ or ‘strongly agreed’, whereas in the intervention group 84.0% ‘somewhat agreed’ or ‘strongly agreed’ (Χ^2^ =9.71, p<0.046, df=4). These data verify that many students increased a desire to receive proper training in nutrition and how to use food as medicine.

Though not statistically significant, five additional items were impacted by the intervention as evidenced by the difference in the level of agreement between the intervention and control groups (Table 2).

**Table 2:** Percentage of Participants Who 'Somewhat Agreed' or 'Strongly Agreed' on Statements Presented at Post-test (n=125) n: sample size; %: percent of individuals in each category For categorical variables, * denotes the significance of the chi-square test comparing the control group to the intervention group.

Questionnaire Statement	Control (n=25)	Intervention (n=100)	Significance p <0.05*
	n (%)	n (%)	
Every day I eat processed foods.	14 (56.0)	49 (49.0)	0.903
Medical school has positively affected my personal nutrition choices.	5 (20.0)	42 (42.0)	0.083
Medical school has positively affected my personal fitness choices.	7 (28.0)	32 (32.0)	0.283
Medical school has positively affected how I plan to incorporate the importance of a healthy diet when advising future patients.	13 (52.0)	83 (83.0)	0.958
Proper nutrition for patient care has been addressed in my coursework to this point.	16 (64.0)	90 (90.0)	0.004*
Proper nutrition can be used to prevent disease.	23(92.0)	100 (100.0)	0.043*
I plan to complete culinary medicine certification following residency.	13 (52.0)	84 (84.0)	0.046*
I plan to explore residency programs that focus on lifestyle medicine.	6 (24.0)	33 (33.0)	0.286

First-year medical students who participated in the nutrition course were at various stages of readiness to change both prior to and after the nutrition course. Data for this element can be found in Table 3.

**Table 3:** Readiness to Change Behavior to Reach a Nutrition-Associated Goal: Before and After Course Completion n: sample size; %: percent of individuals in each category

Stage of Readiness to Change	Prior to Course (n=88)	After Course (n=81)
	n (%)	n (%)
Precontemplation	4 (4.5)	1 (1.2)
Contemplation	22 (25.0)	15 (18.5)
Preparation	35 (39.8)	27 (33.3)
Action	11 (12.5)	20 (24.7)
Maintenance	16 (18.2)	18 (22.2)

Prior to the course, 29.5% of participants were either in precontemplation or preparation, stages that represent low readiness to engage in a behavioral change. After the course, only 19.7% remained in these categories. Approximately twice the percentage of participants were in the “Action” stage of change after the course (24.7%) than were in the group prior to the course (12.5%).

Course effectiveness was analyzed using first-year medical student focus groups. Student feedback regarding the course included numerous positive remarks. Students stated, “I can apply [nutrition] to my life and use it on future patients”, “assignments make us take a critical look at our own lifestyles”, “nutrition has powerful effects on preventative medicine”, and “I'm excited to implement the things I'm learning in my own life and my family's nutrition as well as in my future practice.” Focus group feedback has been utilized to modify the course topic schedule and inclusion of new topics. Students demonstrated an interest in culinary medicine and suggestions to improve physician/patient interactions. A major change was the incorporation of more out-of-class assignments to reinforce and “make real” the impact of course objectives on future practice. Students noted the assignments increased their interest in the material. Students are asked to work independently and in groups to use the material presented in class. Assignments require students to create modified, healthier recipes, complete a personal food diary and dietary analysis, interview a family member to identify nutritional deficiencies and suggest changes, create a personalized physical fitness plan for an obese patient, and complete basic enteral nutrition calculations.

## Discussion

The positive impact of the nutrition course on students’ personal nutrition and their plans to incorporate nutrition into future practice demonstrates the importance of nutrition education within osteopathic medical student education. Students demonstrated an increase in their ability to properly interact with patients regarding nutrition, belief in the power of food as medicine, and a desire to receive further nutrition training. Students who completed the course were also less likely to eat processed foods, more likely to make healthier nutrition choices, and more likely to improve personal fitness choices. Similar outcomes have been demonstrated in other studies, [15] highlighting the need for mandatory nutrition education in all medical professional programs. Implementation of healthier habits will no doubt improve future physicians’ personal lifestyles.

Perhaps even more important than these personal impacts, the nutrition class is affecting how future physicians will discuss nutrition and its effects on health with patients. When a physician can comfortably and knowledgeably discuss nutrition with a patient, it can lead to cost savings through the prevention of diet-related diseases and the reduction of healthcare expenses associated with treating such conditions. Increasing the future physician’s willingness to discuss nutrition and positively affect the weight goals of patients could help curb the growing prevalence of overweight and obese individuals in the population. This study, along with others, has demonstrated that medical students desire nutrition education to feel adequate in nutrition discussions [16,17].

It is important to note that the focus groups have identified that not all first-year medical students have an interest in improving personal nutrition or participating in the discussion of food as medicine with patients. Overall, however, the nutrition class is making a positive impact on the students and providing foundational knowledge for future physicians. As our program produces a high percentage of primary care physicians, nutrition will be part of the physician-patient encounters regardless of personal interest in the topic. Primary care physicians are front-line providers and are often questioned about nutritional advice [18]. As this course increases the importance of nutrition in the future physician’s personal life, perhaps this will increase the likelihood that the physician is willing to discuss nutrition with a patient. A nutrition discussion could provide an early intervention opportunity for many patients as they meet with primary care physicians. As the availability of nutrition education expands across medical school curricula, it is essential to discuss the addition of specific nutrition competencies that should be tested on the board exams, a topic that has been considered for many years [19].

Plans to demonstrate the importance of nutrition education include the expansion of our Lifestyle Medicine Interest Group (LMIG). The LMIG will provide students access to practicing physicians actively incorporating nutrition and the other pillars of Lifestyle Medicine into current practice. Interactions will also provide students an opportunity to practice the skills of proper nutrition in the course and further emphasize the importance of nutrition and other pillars of Lifestyle Medicine.

A key component of the WCUCOM mission is to produce primary care physicians who work in the rural south with underserved populations. These populations of patients could benefit from a physician with nutrition training. Often, rural health clinics do not have dietitian support for basic preventative health clinic visits. The knowledge gained from the nutrition course and further involvement in the LMIG will help our future rural physicians provide preliminary lifestyle interventions for patients and increase the likelihood of referral of patients to virtual nutrition counseling and coaching. In the course, students are exposed to various strategies to initiate the conversation around nutrition and lifestyle. For many patients, the initiation of a conversation is a critical first step in a lifestyle intervention. In addition, specific training allows physicians to provide preliminary guidance to patients when dietitians are not available.

Our findings align with the broader literature, which advocates for the inclusion of nutrition education in medical curricula to better prepare future healthcare providers. By demonstrating the positive impact of the nutrition course on students’ personal and professional development, this study contributes valuable insights into the benefits of comprehensive nutrition training. Future research should explore the implementation of similar courses across different institutions and settings to validate and expand upon these findings. Additionally, longitudinal studies are needed to assess the long-term effects of nutrition education on medical practice and patient outcomes. Further research could also investigate the development of standardized nutrition competencies for medical board exams to ensure consistent and effective nutrition training across medical schools.

Limitations

Despite the study's strengths, such as its examination of the direct impacts of a mandatory nutrition course on first-year osteopathic medical students and the positive outcomes observed, several limitations must be acknowledged. First, the self-reported nature of the surveys may introduce bias, as students might overestimate their engagement with the course material or the impact of the course on their behaviors and attitudes. Second, the study sample consisted solely of students from a single osteopathic medical school, which may limit the generalizability of the findings to other medical schools or healthcare programs. Third, the follow-up period was relatively short, and long-term effects of the nutrition course on students' personal and professional behaviors were not assessed. Lastly, the potential for selection bias exists, as students with a prior interest in nutrition may have been more likely to engage with and benefit from the course. Future research plans address these limitations by incorporating a more diverse sample and additional surveys to allow longer follow-up periods.

## Conclusions

Our findings reflect that the mandatory first-year nutrition course had a positive effect on the osteopathic medical student’s personal dietary choices and plans to incorporate nutrition into future medical practice. These results demonstrate that nutritional education promotes an awareness of the effectiveness of nutritional counseling in disease prevention and management. Furthermore, the course will hopefully encourage future physicians to consider nutritional counseling during their clinical rotations, through residency, and into their independent practice. We believe that the presentation of nutrition in the first year of medical school is critical to expand the number of primary care physicians that promote the importance of nutrition and a healthy lifestyle for patients.

## Appendices

**Table 4:** Nutrition Questionnaire This table represents the survey questionnaire used in the implementation of the nutrition class research project. Each row contains a specific question (in bold) and its corresponding answer options to follow (in italics). The questions cover various aspects of personal nutrition, lifestyle choices, and the impact of medical school on these areas.

Item Number	Survey Question and Answer Options
1.	What is your OMS classification (class of 2024 or class of 2025)?
2.	What is your race?
3.	What is your ethnicity?
4.	Enter your current age in years.
5.	Please rate your agreement with the following statements using the following scale: strongly agree, somewhat disagree, neither agree nor disagree, somewhat agree, strongly agree.
	In my opinion, my personal nutrition is healthy.
	Every day I eat at least three servings of fruit and vegetables.
	Every day I eat at least three servings of lean protein.
	Every day I eat processed foods.
	Every day I eat at least three servings of protein from a meat source.
6.	Please rate your agreement with the following statements using the following scale: strongly agree, somewhat disagree, neither agree nor disagree, somewhat agree, strongly agree.
	Medical school has positively affected my personal nutrition choices.
	Medical school has positively affected my personal fitness choices.
	Medical school has positively affected my sleep habits.
	Medical school has positively affected my mental health.
	Medical school has positively affected my personal lifestyle choices.
7.	Consider the following personal behavioral changes: increasing the number of vegetables and fruit in your diet; increasing the amount of whole plant foods in your diet decreasing the amount of meat-based protein in your diet; increasing the amount of your daily exercise; increasing the amount of sleep you obtain each night; decreasing or ceasing the amount of alcohol or tobacco you use Now, considering those types of changes (others are possible), mark the statement that best represents your readiness to change personal behavior.
	I have never thought about making a behavioral change related to lifestyle choices such as personal nutrition.
	I have thought about making a behavioral change related to lifestyle choices such as personal nutrition, and I will likely make the change in behavior in about 6 months.
	I have been looking into ways to make a behavioral change related to lifestyle choices such as personal nutrition work for me, but I have yet to actually try to change my behavior. I will likely make the change in behavior in about 1 month.
	I made a behavioral change related to my lifestyle choices such as improved personal nutrition less than 6 months ago.
	I made a behavioral change related to my lifestyle choices such as improved personal nutrition and have kept up that change in behavior for 6 months or more.
8.	Please rate your agreement with the following statements using the following scale: strongly agree, somewhat disagree, neither agree nor disagree, somewhat agree, strongly agree.
	Medical school has positively affected how I plan to incorporate the importance of a healthy diet when advising future patients.
	Proper nutrition for patient care has been addressed in my coursework to this point.
	Proper nutrition can be used to prevent disease.
	Proper nutrition can be used to treat disease.
	I plan to incorporate patient education around the importance of a healthy lifestyle into my future practice.
	I plan to incorporate patient education around the importance of proper nutrition into my future practice.
	I would be interested in culinary medicine or food as medicine program track at WCUCOM.
	I would be interested in a lifestyle medicine program track at WCUCOM.
	I plan to explore residency programs that focus on lifestyle medicine.
	I plan to explore residency programs that focus on culinary medicine.
	I plan to complete lifestyle medicine certification following residency.
	I plan to complete culinary medicine certification following residency.
